# COVID-19 Disease in Under-5 Children: Current Status and Strategies for Prevention including Vaccination

**DOI:** 10.3390/vaccines11030693

**Published:** 2023-03-17

**Authors:** Anish Pillai, Anuja Nayak, Deepika Tiwari, Pratichi Kadam Pillai, Aakash Pandita, Sachin Sakharkar, Haribalakrishna Balasubramanian, Nandkishor Kabra

**Affiliations:** 1 Surya Hospitals, Mangal Ashirwad Building, Swami Vivekananda Road, Santacruz West, Mumbai 400054, Maharashtra, India; 2 British Columbia Children’s Hospital Research Institute, 938 West 28th Avenue, Vancouver, BC V5Z 4H4, Canada; 3 Bai Jerabai Wadia Hospital for Children, Acharya Donde Marg, Parel East, Parel, Mumbai 400012, Maharashtra, India; 4 Medanta Super Specialty Hospital, Sector-A, Pocket-1, Amar Shaheed Path, Golf City, Lucknow 226030, Uttar Pradesh, India

**Keywords:** SARS-CoV-2, coronavirus, vaccination, pandemic

## Abstract

Since the coronavirus disease (COVID-19) pandemic hit the globe in early 2020, we have steadily gained insight into its pathogenesis; thereby improving surveillance and preventive measures. In contrast to other respiratory viruses, neonates and young children infected with severe acute respiratory syndrome coronavirus-2 (SARS-CoV-2) have a milder clinical presentation, with only a small proportion needing hospitalization and intensive care support. With the emergence of novel variants and improved testing services, there has been a higher incidence of COVID-19 disease reported among children and neonates. Despite this, the proportion of young children with severe disease has not increased. Key mechanisms that protect young children from severe COVID-19 disease include the placental barrier, differential expression of angiotensin-converting enzyme 2 (ACE-2) receptors, immature immune response, and passive transfer of antibodies via placenta and human milk. Implementing mass vaccination programs has been a major milestone in reducing the global disease burden. However, considering the lower risk of severe COVID-19 illness in young children and the limited evidence about long-term vaccine safety, the risk–benefit balance in children under five years of age is more complex. In this review, we do not support or undermine vaccination of young children but outline current evidence and guidelines, and highlight controversies, knowledge gaps, and ethical issues related to COVID-19 vaccination in young children. Regulatory bodies should consider the individual and community benefits of vaccinating younger children in their local epidemiological setting while planning regional immunization policies.

## 1. Introduction

The ongoing coronavirus pandemic, caused by severe acute respiratory syndrome coronavirus-2 (SARS-CoV-2) infection, has affected over six hundred and fifty million patients worldwide as of January 2023. To date, over 6 million deaths have been reported, and although significant improvement has been made in the vaccination rates, the numbers continue to rise in many countries [1]. The pandemic has significantly impacted the social structure, strained the healthcare systems, and led to catastrophic economic consequences. There has been a rapid surge in the literature regarding epidemiology, pathogenesis, clinical outcomes, and management of adults and children with coronavirus disease 2019 (COVID-19). COVID-19 disease can affect patients across all age groups. Epidemiologic reports have shown that disease severity, need for respiratory support, and complications are much higher in the older age group [2,3,4]. A systematic review in aged-care facilities with COVID-19 disease reported that around one-third of patients needed hospitalization, with a high case-fatality rate of 23% [5]. Infants and young children (under 5 years) have a unique immunophenotype and respond to COVID-19 differently when compared to adults [6,7]. In contrast to adult data, epidemiologic studies in neonates and children have shown that over 90% of those affected have asymptomatic or mild disease [8,9,10]. Despite a recent surge in pediatric and neonatal cases reported, relevant outcomes such as mortality, need for respiratory support, and hospitalization rates have remained lower compared to adults [11,12,13,14,15,16].

The pathogenesis of severe COVID-19 disease includes invasion and replication of the virus in the respiratory tract, followed by viremia, as well as the body’s immune response, the so-called cytokine storm. The immunopathogenesis of disease in children and neonates is unclear and needs further exploration. Various hypotheses have been postulated to explain the low infection rate and severity of COVID-19 disease in young children, such as the role of the immature immune system, paucity of angiotensin-converting enzyme-2 (ACE-2) receptors, passive transfer of antibodies from the mother, presence of cross-reactive antibodies, off-target effects of other live vaccines, and fetal hemoglobin [17,18].

Preventive strategies for COVID-19 disease in young children include protecting pregnant and breastfeeding females to reduce neonatal infections, reducing horizontal spread by strengthening hand hygiene and using face masks, and universal immunization of at-risk individuals. Vaccination has been a significant milestone in controlling the spread of SARS-CoV-2. Various platforms have been used for vaccine development, such as inactivated, live attenuated, viral vector vaccines (replication and non-replication), RNA vaccines, DNA, protein subunit, and VLP (virus-like protein) vaccines [19]. According to a WHO report released in March 2022, 153 vaccines till now have been approved for clinical trials, and 196 vaccines are in preclinical trials. WHO has issued emergency use authorization (EUA) for a total of ten vaccines, including mRNA-based vaccines using selected modified sequences of spike protein genes such as mRNA-1273 (Moderna, Cambridge, MA, USA) and the BNT162b2 (BioNTech, Mainz, Germany/Pfizer, New York, NY, USA); the non-replicating adenovirus vector-based DNA vaccines AZD1222/ChAdOx1 (Oxford Vaccine Group, Oxford, UK/AstraZeneca, Cambridge, UK), JNJ-78436735/AD26.COV2.S (Janssen/Johnson & Johnson, New Brunswick, NJ, USA) and ChAdOx1_nCoV19 (Covishield, Hadapsar, India); inactivated virus vaccines, BIBP-CorV (Sinopharm, Shanghai, China) and CoronaVac (Sinovac Biotech, Beijing, China) Covaxin; protein subunit vaccines, NVXCoV2373 (Novavax, Gaithersburg, MD, USA) and Covovax [19,20]. Although most COVID-19 vaccines are researched and approved for use in adults, an increasing number of vaccine trials are being conducted in children. Only two vaccines have received emergency use permission for children above the age of 6 months: Pfizer-BioNTech BNT162b2 and Moderna mRNA-1273 [21]. There is a paucity of clinical data from phase 3 vaccine trials and a lack of consensus regarding the risk–benefit analysis in infants and younger children [22].

In this article, we discuss the following: (a) epidemiology of SARS-CoV-2 infection in young children; (b) pathophysiology of severe SARS-CoV-2 infection; (c) various mechanisms that support decreased susceptibility in neonates and young children; (d) strategies for preventing perinatal COVID-19; and (e) role of vaccination during pregnancy and in neonates and children.

## 2. Epidemiology of COVID-19

### 2.1. Epidemiologic Patterns across Age Groups

Large observational reports from electronic databases have been added to the literature on the epidemiology of COVID-19 disease in young children. A systematic review by Badal et al. reported that over 80% of children have mild symptoms such as fever, headache, and cough. In this meta-analysis involving 1810 children aged < 21 years, the prevalence of severe disease was 5%, with an overall mortality rate of 0.3% [23]. A multicenter study involving 82 healthcare institutions from 25 European countries reported COVID-19 disease outcomes in 582 children. In this cohort, with a median age of 5 years, around 8% of children needed intensive care treatment [24]. After excluding children and neonates with comorbidities, the risk of needing respiratory support and intensive care was much lower [25]. A systematic review by Bhuiyan et al. included 1214 children under the age of 5 years with laboratory-confirmed COVID-19 infection. Half of the young COVID-19 cases in this cohort were infants, and nearly half were asymptomatic. Only 7% of cases needed intensive care unit (ICU) care, and one death was reported [26]. Dona et al. reported the outcomes of 216 infants less than 90 days old affected with COVID-19 disease from 35 Italian institutions. Only 2.3% of infants needed intensive care unit admission, and all infants recovered from the illness [27]. A population-based study from the UK reported 66 neonates with confirmed SARS-CoV-2 infection (incidence 5.6 per 10,000 live births) required hospital admission. Trevisanuto and colleagues performed a systematic review of 44 newborns with confirmed SARS-CoV-2 infection. They found that most neonates were asymptomatic or had mild symptoms, with a good overall prognosis [9]. Although vertical transmission of SARS-CoV-2 has been reported, it is rare. According to a live systematic review including 14,271 babies born to COVID-19-positive mothers, only 1.8% of the babies tested positive for SARS-CoV-2. Based on placental or cord blood testing, only 7 babies had confirmed vertical transmission. Around 18% of the neonates who tested positive were symptomatic; however, many babies had overlapping symptoms due to prematurity [28]. Another systematic review showed that only 2% of neonates born to 1100 pregnant women with COVID-19 disease required admission to the neonatal intensive care unit (NICU) [29]. A large cohort of pregnant women with perinatal SARS-CoV-2 infection showed that 2.2% of newborns tested positive, with no increase in mortality or proportion of babies needing mechanical ventilation [30].

### 2.2. Epidemiologic Patterns Witnessed across Time Periods

With the emergence of new variants beyond the first COVID-19 wave, there has been a higher incidence of COVID-19 disease reported in pregnant women, children, and neonates. Despite this rise in incidence, the number of children with severe illness has not increased [31,32,33,34]. A recent report from Poland showed that during the second COVID-19 wave, 30% of affected children were infants, which increased from 10% during the first wave. Around 98% of the infants had mild disease, and none had severe disease [15]. A nationwide American study reported outcomes for under-5 children affected by the Omicron variant compared to those infected by the Delta variant. Most infections were mild, with hospitalization rates being 1% with Omicron and 3% with the Delta variant. Only 0.14% of children with Omicron-variant disease needed ICU admission, compared to 0.43% of children affected with the Delta variant [35]. Reports from the United States suggested that the Delta variant was more transmissible in indoor situations and households, which may lead to higher attack rates among children [36]. Overall, in the epidemiological analysis of COVID-19 in infants during the Delta variant era, there was a significant increase in pediatric cases. This difference in epidemiologic patterns could be possibly related to improved testing and reporting of disease, less stringent isolation measures, mutations in viruses, and aggressive vaccination drive.

The actual incidence of COVID-19 in neonates and infants continues to be underestimated, mainly due to asymptomatic cases and a lack of testing. Studies reporting higher morbidity and mortality in neonates and infants have not accounted for pre-existing comorbidities or used an arbitrary definition for diagnosing severe disease [27]. Epidemiologic data seems promising to propose that neonates and young children may continue to evade severe COVID-19 disease during the upcoming period.

## 3. Pathophysiology of Severe SARS-CoV-2 Disease

**Figure 1:**
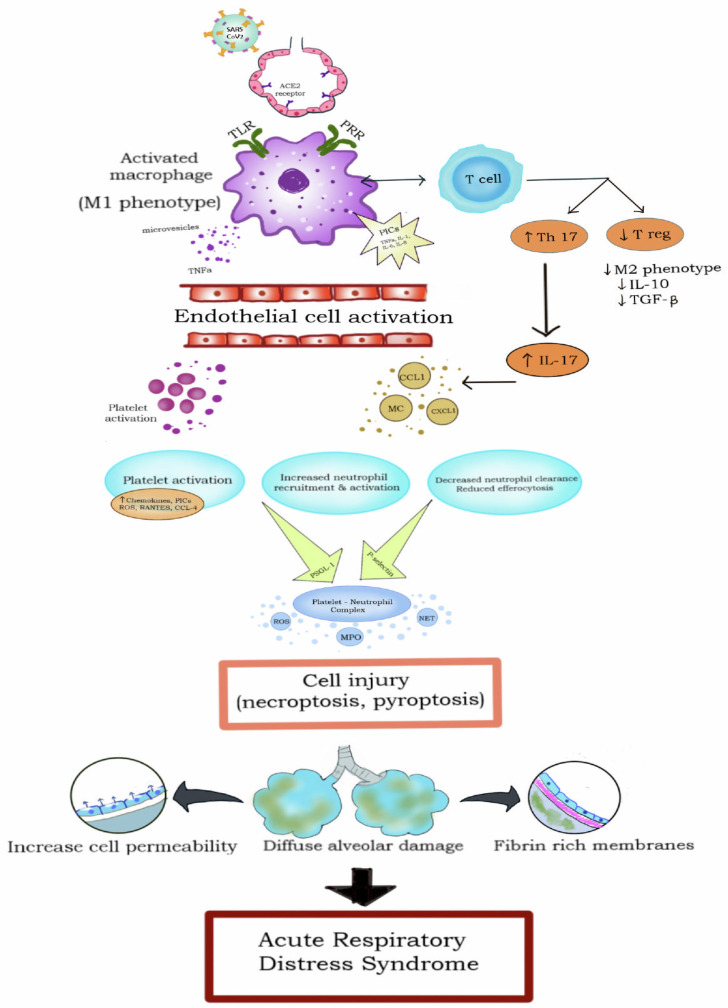
Pathophysiology of acute respiratory distress syndrome in SARS-CoV-2 infection. SARS-CoV-2, severe acute respiratory syndrome coronavirus 2; ACE, angiotensin-converting enzyme; Th, T-helper cell; T-reg, T-regulatory cell; IL, interleukin; TLR, toll-like receptor; PRR, pathogen recognition receptor; PIC, proinflammatory cytokine; TNF, tumor necrosis factor; TGF, transforming growth factor; ROS, reactive oxygen species; RANTES, regulated on activation, normal T cells expressed and secreted; NET, neutrophil extracellular traps; MPO, myeloperoxidase.

The crucial mediator in the pathogenesis of severe SARS-CoV-2 infection is the body’s immune response [37,38]. Histopathologic data shows that acute respiratory distress syndrome (ARDS) secondary to SARS-CoV-2 is characterized by diffuse alveolar damage, inflammatory cells, fibrin-rich hyaline membranes, increased permeability of the epithelium, and interstitial edema, leading to disturbed gas exchange and eventually hypoxic respiratory failure [39,40,41]. Entry of SARS-CoV-2 leads to the increased polarization of activated macrophages to the highly inflammatory M1 phenotype. Activated macrophages and T-cell interaction lead to an increased Th17:T-reg ratio [42,43]. Activated macrophages also secrete many proinflammatory chemicals (PICs), including TNF-alpha, IL-1, IL-6, and IL-8 [44,45]. PICs upregulate the nuclear factor kappa light-chain enhancer of activated B cells (NF-κB), leading to the activation of endothelial cells. Rapid infiltration of activated neutrophils can be a source of toxic neutrophil extracellular traps (NETs) [46]. Platelet activation can augment neutrophil responses, including phagocytosis and the production of oxygen radicals [47]. Platelet-neutrophil complex releases reactive oxygen species (ROS), myeloperoxidase, and NET; all of which contribute to cell death by pyroptosis or necroptosis. The pathophysiology of lung injury related to SARS-CoV-2 is described in Figure 1.

## 4. Mechanisms Protecting Neonates and Children

Several hypotheses have been put forth to explain the lower incidence and severity of COVID-19 disease in young children; however, many of these mechanisms still need to be better understood. The evidence and knowledge gaps regarding these hypotheses are described below and summarized in Table 1.

(i) ACE-2 receptor expression: The SARS-CoV-2 attaches to the ACE-2 receptor in the epithelium via the S-protein, similar to SARS-CoV-1 [79]. ACE-2 is found in multiple sites, including epithelial cells of the oral, nasopharyngeal, and oropharyngeal mucosal epithelium; alveolar epithelium; endothelium of blood vessels and the heart; renal tubules; and small intestinal enterocytes [80]. Virus entry into the cells is facilitated by transmembrane protease serine 2 (TMPRSS2), and cathepsin L-mediated cleavage of ACE-2 [81]. Wang et al. studied gene expression in various age groups and noted that a higher proportion of alveolar epithelial cells expressed ACE2 and TMPRSS2 in adult lungs [82]. ACE-2 expression was deficient in normal newborn lungs. Additionally, very few cells express both ACE-2 and TMPRSS2 (double-positive cells) in young children [54,83]. This could explain the lower susceptibility of neonates; however, there is conflicting data regarding this topic. ACE-2 receptors decrease in the elderly population, which has a higher susceptibility to severe COVID-19 disease [84].
(ii) Placental barrier: Epidemiologic studies have ascertained that transplacental transmission from an infected mother to her baby is possible but rare [10,17,48,49,50]. The continuous cell layer over the placental surface and dense actin filament network over the brush border serve as a physical barrier to the transmission of pathogens. In addition, the placenta also secretes type 3 interferon miRNAs, which act as antiviral compounds [85,86]. None of the villous stromal cells or Hoffbauer cells (placental macrophages) express ACE2; also, expression of TMPRSS2 is weak in the syncytiotrophoblast. This may explain the low risk of congenital infection in SARS-CoV-2 disease. A study by Algarroba et al. [51] demonstrated the localization of corona virions in the placental syncytiotrophoblast of the placenta in a COVID-19-positive mother, although the neonate tested negative for COVID-19. Similar reports suggest that symptomatic neonatal infection remains uncommon despite placental infection [87,88].
(iii) Immature immune system: Immune responses in fetal life are curbed so that they can co-exist with the semi-allogenic maternal immune system. In the neonate, the majority of T cells are immature. CD45RA, a marker expressed on naive T cells, is found in 90% of cord blood T cells, compared to 40% of adult T cells [58,89]. A large proportion of fetal CD4 cells develop into CD25+FOXP3+ regulatory T cells (T-reg cells). T-reg cells play an essential role in the downregulation of immune responses and promote tolerance [90,91]. The monocyte-macrophage system during infancy has an impaired ability to produce inflammatory cytokines [92]. The benefits of immune tolerance have been well described in viral infections such as hepatitis B (HBV), where acute hepatitis is rarely seen in the neonatal period [93].

**Figure 2:**
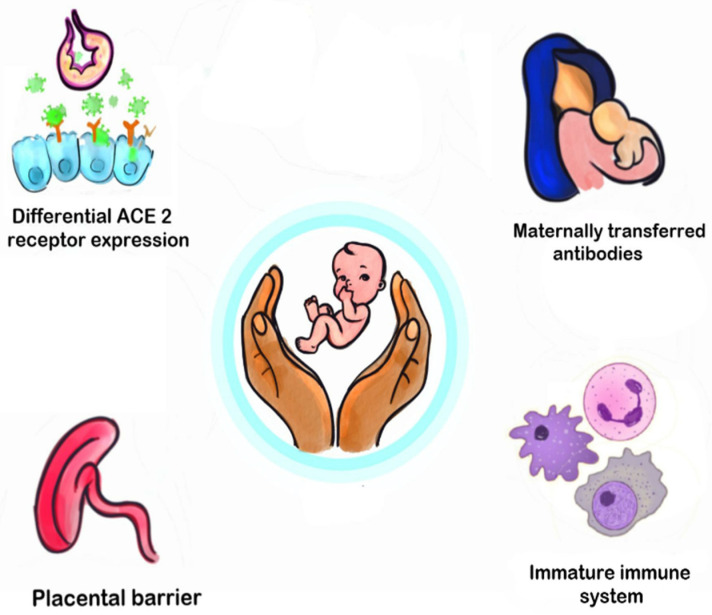
Key mechanisms by which young children resist SARS-CoV-2 infection. ACE, angiotensin-converting enzyme.

At the same time, the immature immune system is highly prepared to react to novel pathogens, such as SARS-CoV-2. Exposure to a novel pathogen releases polyreactive IgM antibodies, independent of antigen exposure. These antibodies have wider reactivity and the ability to rapidly induce further production of natural antibodies [94]. Moreover, the predominance of CD27^dull^ memory B cells after novel antigen exposure leads to a faster reaction [60]. This balance between immune tolerance and preparedness to face unique pathogens is a vital strength of neonatal immunity.

(iv) Maternal immunoglobulins: Maternal immunoglobulins are crucial in the first few months after birth. In a case series by Zeng et al., including mothers with recent COVID-19 disease, 5 out of 6 mothers had high IgG titers > 50 AU/mL (reference range < 10 AU/mL). All five neonates born to these mothers had IgG titers > 50 AU/mL but lower than maternal titers, suggesting transplacental transfer of antibodies. Two infants also had positive IgM titers, suggesting possible fetal antibody production 95. Other studies have also supported the mother-to-child transmission of COVID-19 antibodies [96,97,98]. The IgG transplacental transfer ratio is higher when the first maternal-positive PCR is 60–180 days before delivery compared with <60 days [98]. Typically, maternal antibodies decline rapidly after the first 3–6 months of life; however, there is limited data on how long the maternal COVID-19 IgG antibodies confer protection to the newborn.
(v) Human Milk: Bioactive and cellular components of breastmilk together modulate the maturation of the neonatal immune system [99,100]. Pace and colleagues tested 37 milk samples and 70 breast swabs from 18 women recently diagnosed with COVID-19. SARS-CoV-2-specific IgA and IgG antibodies were found in all milk samples. No milk samples detected SARS-CoV-2 RNA [63]. Other reports have also shown the presence of secretory IgA and IgG in the breastmilk of SARS-CoV-2-infected mothers [64,65,101]. SARS-CoV-2 RNA was found in several breast swabs, highlighting the importance of continued efforts towards hygiene measures [63]. Based on current evidence, the benefits of breastmilk feeding far outweigh the theoretical risk of virus transmission via breastmilk.
(vi) Immature lung: Alveologenesis and lung maturation is a continuous process for up to 7–8 years [102]. Animal studies show that pulmonary endothelial cells in neonates can better preserve the barrier function post-endotoxin administration compared to adults [70].
(vii) Melatonin: Melatonin is anti-inflammatory and can protect against ARDS and hemorrhagic shock during viral infections [103,104]. Higher melatonin levels may contribute to milder disease course in young children. Results from the ongoing randomized trials to test the efficacy of melatonin as a prophylactic agent are awaited [73,74].
(viii) Live vaccine effect: Live vaccines such as BCG can have immunomodulatory properties extending beyond the usual protection against the target pathogens [105,106,107]. Epidemiologic data suggest variances in the prevalence and severity of COVID-19 disease in countries with different BCG vaccination policies [107,108,109]. Clinical trials testing this hypothesis are ongoing [78]. However, data from the second pandemic wave suggests high prevalence and mortality even in countries that give routine BCG vaccination [110,111].
(ix) Microbiota: Microbiota plays a crucial role in immune regulation and inflammation. Studies have reported differences in microbiota between patients infected with COVID-19 and healthy adults [67,68]. A pilot study including 15 patients with COVID-19 infection showed enrichment of opportunistic pathogens (Streptococcus, Rothia, Clostridium, and Actinomyces) and depletion of beneficial commensals (Faecalibacterium, Bacteroides) at all timepoints during hospitalization [68]. The summary of fundamental mechanisms that may possibly confer protection to young children from COVID-19 disease is listed in Figure 2.

## 5. Strategies for Preventing Perinatal COVID-19

### 5.1. General Measures

Neonates and young children may have milder COVID-19 disease; however, they act as an essential source of transmission due to close proximity with many generations [112]. Separation of newborns and parents, limited visiting policies in intensive care units (ICU), and closure of schools were common strategies used in many countries as emergency measures to control the rapid surge of COVID-19 cases. However, these were urgent measures taken by policymakers with little evidence to support them and may come with their own set of adverse effects. Separation of the mother and newborn disrupts skin-to-skin care, reduces breastfeeding, and increases maternal anxiety and stress [113,114]. School closure has increased screen time and behavioral concerns such as higher anxiety in children [115]. Other effects such as insomnia, emotional disturbance, irritability, sleep and appetite disturbances, negative eating habits, obesity, and impairment in social interaction have also been reported in children [116]. Protecting the mother during pregnancy and the postpartum period is a crucial step in reducing the spread to neonates. Early observational studies have shown that patient triage at hospital arrival, universal testing of pregnant women with nasopharyngeal swabs, use of face masks, hand hygiene and personal protective equipment by hospital staff, and infection control practices can minimize the horizontal spread of the virus [117,118,119,120,121,122]. The general strategies (apart from vaccination) for preventing SARS-CoV-2 spread are summarized in Table 2.

### 5.2. COVID-19 Vaccines

The widespread vaccination approach has been a game-changer in reducing the spread as well as the severity of COVID-19 disease in adults [151,152]. Vaccinating pregnant women is a vital strategy to reduce the risk of SARS-CoV-2 transmission to the fetus and newborn baby. Although initial COVID-19 vaccine clinical trials excluded pregnant and lactating women, substantial data from observational studies have since reported safety and benefits for use in this population [153,154,155,156]. Preliminary clinical data has shown that COVID-19 vaccination is safe and has over 90% efficacy in preventing infections in older children and adolescents [157]. The objectives of a vaccination program in children include reducing the incidence of disease, decreasing the burden of severe disease, lowering transmission rates, and preventing long-term complications. These benefits must be balanced with the safety profile based on the available clinical evidence. The efficacy and long-term safety data of COVID-19 vaccines in younger children (<5 years) need further evaluation. 

#### 5.2.1. COVID-19 Vaccines Applicable during Pregnancy

(i) Vaccine type: Vaccines that have the most published data regarding their use in pregnancy are mRNA vaccines such as Pfizer-BioNTech’s COVID-19 vaccine and Moderna’s COVID-19 vaccine, and recombinant vector-based vaccines such as Oxford/AstraZeneca and Johnson & Johnson’s/Janssen. Other vaccines such as the protein subunit vaccine (Novavax) and inactivated-virus vaccine (Covaxin) are also considered safe in pregnancy; however, there is limited clinical data in this regard [158,159,160,161,162,163]. Current recommendations suggest that the COVID-19 vaccine, including the primary series or booster, can be given in any trimester along with routine vaccines during pregnancy, such as influenza and Tdap [153,156,158].
(ii) Vaccine Efficacy—benefits to newborns: There is adequate evidence to support the claim that pregnant women produce a robust antibody response post-vaccination [164,165]. Vaccine-induced antibody titers may be similar to those produced in non-pregnant women, with antibody production starting two weeks from the first dose [166]. Benefits of the vaccine for the mother include reduced risk of infection, lower maternal hospitalization, and need for intensive care admission [167,168]. Vaccination in pregnant women also benefits the neonate due to the passive transfer of antibodies. The placental transfer of antibodies depends on the maternal antibody titer, vaccination timing, and delivery interval. A recent prospective cohort study found that 57% of infants with detectable antibodies against COVID-19 at six months of age were born to vaccinated pregnant females, compared to only 8% of infants with detectable antibodies in infants born to individuals who had COVID-19 illness during pregnancy [166]. Studies have shown that vaccination with the mRNA COVID-19 vaccine series during pregnancy may protect babies up to 6 months of age from severe COVID-19 disease and hospitalization [156]. Third trimester (27–31 weeks) vaccination is associated with higher neonatal anti-SARS-CoV-2 antibody levels, and the early third trimester may be the optimal timing for a booster dosing [169]. Passive immunity can protect neonates against severe COVID-19 disease; however, studies are still underway to determine exactly how these antibodies work [170,171].
(iii) Vaccine Safety—for mother and fetus: Data from vaccine safety monitoring systems have not found any safety concerns after mRNA COVID-19 vaccination during pregnancy or lactation for females or their babies. Observational data from various countries have shown that vaccination with the mRNA COVID-19 vaccine during pregnancy before 20 weeks was not associated with an increased risk of complications, including preterm delivery, stillbirth, postpartum sepsis, or postpartum hemorrhage [153,154,172,173]. There was no association between COVID-19 vaccination in pregnant females with congenital anomalies [174]. A recently published systematic review suggested a possible reduction in the risk of stillbirth in the vaccinated population by 15% (pooled OR 0.85; 95% CI 0.73–0.99). The risk of adverse maternal outcomes such as spontaneous abortion, placental abruption, postpartum hemorrhage, increased maternal mortality, intensive care unit admission, or neonatal adverse outcomes such as preterm birth, fetal growth restriction, and NICU admission (*p*  >  0.05 for all) was not increased in vaccinated women [160].
(iv) Side effects: The side effects related to the COVID-19 vaccine during pregnancy include injection site pain, myalgia, headache, and fatigue, which are similar to those described in the non-pregnant population [153,175]. Prospective studies comparing vaccinated pregnant women matched with vaccinated female non-pregnant controls have reported minor differences in the reporting of side effects in the two groups, but the overall frequency of complaints was similar [176,177]. COVID-19 vaccination is a safe and effective method for reducing disease burden and severity in pregnant women and their babies.

#### 5.2.2. COVID-19 Vaccines Applicable to Young Children

The justification for universal COVID-19 vaccination in healthy children under five years of age is an ongoing debate. The risk–benefit ratio for COVID-19 vaccination in children is more dynamic and intricate than in adults, and global vaccination guidelines are not well established in younger age groups. Considerations to decide the role of vaccines in young children (<5 years) include vaccine safety, prevention from long-term consequences such as multisystem inflammatory syndrome in children (MIS-C) and long COVID-19 disease, community spread, availability, and costs of vaccination.

(i) Vaccine type: The US FDA recently amended the emergency use authorizations (EUAs) of the Moderna and Pfizer-BioNTech COVID-19 vaccines to include use in children down to 6 months in December 2022 [21]. The vaccination schedule for children as per the CDC is summarized below [178].

The Moderna COVID-19 mRNA-1273 vaccine schedule:

- 6 m–5 years age: Two doses of 25 µg (0.25 mL) 4 weeks apart. Booster dose at least 2 months after the primary series (updated/bivalent).
- 6–11 years age: Two doses of 50 µg (0.25 mL) 4–8 weeks apart. Booster dose at least 2 months after the primary series dose (updated/bivalent).
- The Pfizer-BioNTech BNT162b2 vaccine schedule:
- 6 m–4 years age: Three doses of 3 µg (0.2 mL) first and second dose 3–8 weeks apart; third dose 8 weeks after the second dose. Third dose can be updated/bivalent vaccine. No booster dose recommended.
- 5–11 years age: Two doses of 10 µg (0.2 mL) 4–8 weeks apart. Booster dose at least two months after the primary series (updated/bivalent).

Other vaccines being evaluated for use in children include Gam-COVID-Vac (Sputnik, Moscow, Russia), Covaxin, ZyCov-D (Zydus Cadila, Ahmedabad, India), Covovax, and Johnson & Johnson’s/Janssen.

(ii) Vaccine Efficacy: Observational studies post-introduction of COVID-19 vaccines in children have reported variable effectiveness of the primary series, ranging between 25% and 50% [179,180,181,182]. Unvaccinated children have two times higher cumulative hospitalization rates when compared to vaccinated children [183]. A nationwide cohort from Chile, including 490,694 children aged 3–5 years, showed vaccine efficacy of 38% (95% CI, 37–40) against symptomatic COVID-19, 65% (95% CI, 50–75) against hospitalization, and 69% (95% CI, 19–88) against ICU admission [184]. Moreover, data in the adolescent age group have shown that two doses of mRNA vaccine has an effectiveness of 91% for MISC-C [185]. In a large observational study including nearly 200,000 children and adolescents using the database in Israel, Amir and colleagues estimated rates of confirmed SARS-CoV-2 infection according to Pfizer-BioNTech vaccination status in children aged 5–15 years. The infection rates were more than two times lower in children after the second vaccine dose compared with same-aged children after the first dose. Furthermore, the infection rates in adolescents were lower 2–8 weeks after the booster dose, compared to 3–7 days after booster dose [186]. Thus, booster doses in adolescents increase protection against infection.
(iii) Vaccine safety: Children who received the Pfizer-BioNTech COVID-19 vaccine reported mostly mild local (86.2%) and systemic (66.6%) reactions; no other serious adverse events were found to be associated with vaccination [187]. Febrile seizures post-COVID-19 vaccines were rarely reported in young children, and incidents happened with similar rates for both mRNA vaccines (Pfizer and Moderna COVID-19) [188]. Severe allergic reactions such as anaphylaxis are infrequent in children post-COVID-19 vaccination [189]. Among children 5–11 years, there were no cases of myocarditis. Rare cases of myocarditis and pericarditis have been reported after vaccination in older children and adolescents who received the mRNA vaccine. In these reports of myocarditis, the incidence was found to be higher after the second dose of the mRNA vaccine (Pfizer-BioNTech) [190]. Thrombosis with thrombocytopenia syndrome (TTS) has been reported in young adults following adenoviral-vector vaccines; however, there are no such reports in children [191].
(iv) Side effects: According to an extensive online survey, around 50% of children reported side effects post-vaccination. The most commonly reported symptoms were injection site pain, fatigue, fever, and headache. There were no serious adverse events, and symptoms improved within 1–3 days after vaccination [192]. Another study from Saudi Arabia reported that 60% of adolescents had at least one side effect post-vaccination. The common side effects were injection site pain, fever, fatigue, headache, and nausea. Side effects were more common after the second dose [193]. The side-effect profile of the COVID-19 vaccine is mild and similar to other flu vaccines. Evolving observational reports after vaccination in younger children and infants will add to the safety data regarding COVID-19 vaccines.

#### 5.2.3. Knowledge Gaps and Controversies Related to Pediatric COVID-19 Vaccination

(i) Vaccine dose and interval: The lower antigen doses suggested for the pediatric population are based on small trials. A post-implementation trial of the 10 µg dose of Pfizer-BioNTech vaccine in 5–11-year-old children showed an effectiveness of less than 50%, with immunity rapidly waning within 3 months after vaccination [180]. The dose of 3 µg recommended for children aged 6 months to 5 years is mainly based on tolerability data, with limited evidence on efficacy [178]. Regarding the interval between two doses, studies in adults have evaluated different COVID-19 vaccine regimens and their immunological and epidemiological impact. Reports suggest that delaying the second vaccine dose up to 12 weeks may improve longer-term immunity [194,195,196]. However, data regarding optimal dosing and intervals in young children are lacking, and recommendations are extrapolated from adult and adolescent data.
(ii) Age cutoff: The lower age cutoff for the primary vaccination series varies between countries, ranging from 6 months to 12 years. This is related to the paucity of data regarding vaccine safety and effectiveness in younger children, optimal dosage schedule, and lower risk of severe illness. The Center for Disease Control and Prevention (CDC) in America recommends universal COVID-19 vaccination above the age of 6 months [178], China provides vaccination to children above 3 years of age [197], Australia recommends routine COVID-19 vaccination above 5 years [198] or children between 6 months and 5 years meeting the high-risk criteria, whereas in many countries such as India only children above 12 years are eligible for the vaccine [199].
(iii) Mixed vaccine strategy: A mixed-vaccine strategy was initially proposed in view of limited vaccine supplies. A recently published single-blinded, randomized, non-inferiority trial from the UK aimed to determine the safety, immunogenicity, and reactogenicity of heterologous primary vaccination including mRNA, viral vector, and protein adjuvant vaccine, included 1072 adults and found that heterologous second dosing with m1273 (Moderna) increases systemic reactogenicity compared with homologous schedules [200]. Ongoing trials are being carried out in the adult population to explore heterologous vaccination; however, extrapolating this to the pediatric population is probably not justified [201]. For the primary vaccination series in adults and for all doses in young children, using the same product and brand is preferable [178].
(iv) Immunocompromised children: Due to the complex pathogenesis of infection and altered immune response, immunocompromised children are a unique cohort at higher risk of severe COVID-19 disease. The role of the COVID-19 vaccine is crucial in this cohort. Although the initial COVID-19 vaccine trials excluded immunocompromised children, the general expert consensus recommends that non-live vaccines can be safely administered to such patients [202]. The CDC also suggests that immunocompromised children must receive an additional primary dose of the COVID-19 vaccine as they may not respond adequately to the standard two-dose series. The Australian Technical Advisory Group on Immunization has identified children at higher risk of severe COVID-19 disease such as those with primary/secondary immunodeficiency, use of immunosuppressive drugs, bone marrow transplant recipients, complex cyanotic heart disease, chronic lung disease, and type 1 diabetes mellitus [203]. However, many areas need further evaluation, such as optimal dosage, timing of vaccination, and consideration regarding the withholding of immunosuppressive medications after vaccination [204].
(v) Variants of concern: Studies have shown that mRNA vaccines effectively reduce infection and transmission of SARS-CoV-2 variants of concern (VOC), such as the Alpha, Beta, and Delta variants [205,206]. The Omicron variant described in November 2021 contains around 50 mutations, 32 of which are located in spike protein, which is the primary target for mRNA vaccines. This creates an unstable binding affinity of the receptor domain to ACE2 receptors, hence making mRNA vaccines less effective [207,208]. Since the immune response in children is very much distinct from the adult population, it is necessary to demonstrate VOC susceptibility to neutralization antibodies in the pediatric population. Data published from Hong Kong suggested that children had lower neutralizing antibody titers against the Omicron variant than those against the ancestral viral strains [209]. Although booster doses may improve the neutralizing antibody levels for VOC, they remain lower than those for the original strain [210]. These findings suggest that the pediatric age group may be more susceptible to vaccine breakthrough infections caused by Omicron or a newer VOC. Repeated mutations and the appearance of new VOC can affect vaccine efficacy in children and warrant continuous surveillance of the risk–benefit balance [211].

#### 5.2.4. Ethical Issues Related to COVID-19 Vaccine

(i) Vaccine Hesitancy: Early reports suggested that a significant portion of the population including healthcare workers were hesitant to take vaccination [212,213]. There are noticeable racial/ethnic differences in the likelihood of vaccine uptake in the general population [214]. Current immunization regimes have shown modest efficacy and rapidly waning protection in 5–11-year-old children post-vaccination [180]. An online survey of 411 female guardians of children aged 1–4 years reported determinants of COVID-19 vaccine hesitancy across diverse ethnic and geographic backgrounds. Only 31% of parents expressed their intention to vaccinate their child; 23% of parents were unsure; and 46% intended not to vaccinate. The main reasons for vaccine hesitancy were concerns about side effects in young children, the hasty nature of vaccine approval, and distrust in pharmaceutical companies. For parents who were unsure about vaccination, there was a belief that children were not susceptible to infection and that the vaccine was not effective against new variants. Other published studies have also reported that lack of high-quality clinical evidence, accelerated vaccine approvals, and misleading information over social networking sites were significant factors for resistance to COVID-19 vaccines [215,216,217]. There is an urgent need for improved public health messaging, transparency regarding vaccine trials and approval, community outreach programs, and increased involvement of pediatricians or family physicians to address family concerns [218].
(ii) Vaccine mandates: Healthcare institutions were the first to implement vaccine mandates for healthcare professionals (HCP). Vaccine mandate has been previously practiced in the healthcare sector, as HCPs are required to receive immunization against diseases such as influenza, pertussis, and hepatitis B [219]. The initial voluntary COVID-19 vaccination drives did not achieve the coverage necessary to protect the community, and outbreaks were frequent. Many institutions and regulatory bodies have since implemented mandates, with the vision to pursue the health and well-being of as many individuals as possible and to avoid harm. For example, the justification for vaccination mandates in schools may be the risk of frequent school disruption and the well-being of other students. Restricting the use of public transport, entry to closed public spaces (theatres, malls), and denying entry to countries have been measures to implement vaccine mandates. The ethical principle of autonomy conflicts with vaccination mandates, as it can override individual choices [220]. Measures such as effective dissemination of vaccine safety data with a mass-educational approach; making school vaccination camps accessible and safe; availability of pediatricians and emergency facilities for those who need them; and providing positive reinforcement for participants in the vaccine programs will improve compliance rates. Creating policies that respect and accommodate the autonomy of the parent and child remains the key method for moving forward.
(iii) Vaccine Research: The norm for performing vaccination research involving children is to commence clinical trials only when a vaccine has been shown to be safe and effective in adults. However, this will lead to a delay in vaccine approval, which could impact the global measures to curb the pandemic. Conducting vaccine trials in children has many issues. The trials need to involve children living with high-risk health conditions and disabilities (whether neurological, genetic, physical, developmental, or emotional), as they are the population most likely to benefit from the vaccine. Whether future vaccine trials should be placebo-controlled or not is another matter of controversy. This is relevant in countries where emergency approval is granted for certain vaccines, as it is difficult to justify continuing with a placebo-controlled trial in the context of the current pandemic [221,222]. For vaccination research in children, the guiding principles should be transparency in data reporting (broken down raw data from clinical trials), inclusiveness (of high-risk children), and external validity.

#### 5.2.5. Implementation of Childhood Vaccine Program

Further research is needed to explore the effectiveness of vaccines in younger age groups, cost–benefit analysis, and their role in children previously infected by the disease. Simultaneously, surveillance regarding disease severity across children of various age groups is essential. The role of universal immunization of healthy children needs to be evaluated in the epidemiological milieu of each country to plan immunization policies. We suggest the following measures to aid the implementation of a vaccination program for children [199].

(i) Prioritization: Vaccination of younger children should be considered only if the region/country has achieved high immunization coverage rates for older children (6–11 years) and adolescents. Initially, children aged 2–5 years with high-risk conditions should be immunized on a priority basis. The next priority could be for children living with high-risk individuals. Lastly, children less than 2 years should be included. An age de-escalation approach should be used once sufficient vaccine coverage has occurred in higher age groups.
(ii) Involvement of Pediatricians: Pediatricians and family physicians are an integral part of childhood vaccination programs, as they have good rapport with the child and family. Families may be most comfortable getting the COVID-19 vaccine in Pediatric clinics. The protocols for cold chain maintenance, recognition and management of vaccine-related adverse events, and disposal of biomedical waste are well established in such centers.
(iii) School-based vaccination program: Conducting school- or college-based vaccination camps is a quick and effective tactic to maximize coverage; however, these centers should have adequately trained personnel to handle the vaccine administration and adverse events. This should be voluntary with the informed consent of the child and guardian. These centers should have facilities for quick transport to referral hospitals if required.
(iv) Mandates: With current evidence, less intrusive measures should be used to encourage voluntary vaccination against COVID-19 in children. Current risk–benefit analysis does not support vaccine mandates for children as an ethically justifiable option.
(v) Surveillance: An ongoing active and passive surveillance system must be set up to monitor and report any adverse events related to the vaccine. This will also provide long-term data on the safety of these vaccines for children.

## 6. Conclusions and Future Directions

Recent epidemiologic data have reaffirmed that neonates and young children have succeeded in circumventing severe SARS-CoV-2 infection, compared to other age groups. The placental barrier, differential expression of ACE-2 receptors, immature immune response, and transfer of maternal antibodies seem to be key reasons for this protection. However, the exact mechanisms still need to be better understood. With increasing vaccination coverage in pregnant women and older children, the global burden of severe disease has been reduced. Vaccinating younger children has helped to briefly improve their antibody levels; however, data are lacking regarding the long-term safety and efficacy of vaccines in younger children. Current evidence does not support mandatory COVID-19 vaccination programs as an ethically justifiable policy option for children. Countries should deliberate regarding the individual and community benefits of vaccinating children in their unique epidemiological context while planning their immunization policies. Future studies should focus on the long-term safety and benefits of the COVID-19 vaccine in under-5 children, with a special focus on high-risk children.

## Figures and Tables

**Table 1:** Evidence and knowledge gaps regarding various proposed mechanisms protecting neonates from COVID-19 disease.

Mechanism	Evidence (Direct/Indirect)	Knowledge Gaps
Placenta—barrier for preventing viral transmission	Low rate of vertical transmission [10,48,49,50] Neonate not infected despite detection of virion in syncytiotrophoblast [51] ACE-2 expression in syncytiotrophoblast towards the stromal side [52,53]	Impact of gestational age at which mother acquires infection on vertical transmission Risk factors that promote virus transmission to the fetus
Receptor expression—decreased attachment of virus	Reduced expression of ACE-2 and TMPRSS2 in children [54] Less co-localization of ACE-2 and TMPRSS2 [54]	Role of drugs in manipulating ACE2–viral interaction
Immature Immune system—immune tolerance and faster response to novel antigens	Abundance of naive T-cells [55] Regulatory T cells downregulates the immune response [56,57] Immature B-cells produce polyreactive antibodies [58,59] Predomination CD-27 dull memory B cells [60]	Efficacy of immune modulators in children with severe COVID-19 disease
Maternal Immune transfer—transfer of protective antibodies	Transplacental transfer of SARS-CoV-2 IgG antibodies to the baby [61,62]	Factors influencing antibody transfer from mother to baby
Breast milk—transfer of antibodies and live cells	Secretory IgA and IgG antibodies against SARS-CoV-2 found in breastmilk of mothers with history of infection [63,64,65]	Duration and degree of protection by antibodies transferred through breastmilk
Microbiota—presence of beneficial commensal microbes	The neonatal microbiome influences immunological, endocrine, and neural pathways [66] Altered microbiota in patients with COVID-19 [67,68]	Role of probiotics or concurrent antibiotic use on the severity of COVID-19
Developing lung—better healing and less fibrosis	Children recover better from ARDS compared to adults [69] Neonatal pulmonary endothelial cells have better preservation of the pulmonary barrier function [70]	Role of surfactant and steroids in neonates with COVID-19 pneumonia
Melatonin-anti-inflammatory and antioxidant	Melatonin peak levels decrease with age [71,72]	Ongoing trials evaluating effect of melatonin on COVID-19 pneumonia [73,74] Normative data on melatonin levels lacking
Vaccine off-target effect—trained immunity	BCG vaccine can confer protection against viruses [75,76,77]	Trial to test this hypothesis ongoing [78]

**Table 2:** Preventive strategies to reduce COVID-19 transmission.

Strategy	Evidence (Direct/Indirect)	Recommendations
Universal screening for COVID-19 disease in pregnant women	SARS-CoV-2 infection in pregnancy is associated with higher morbidity and mortality [123,124,125]. Around 14–27% women tested positive for SARS-CoV-2 with universal screening during admission for delivery [126,127,128].	In areas with high prevalence of COVID-19 disease, all pregnant women hospitalized for delivery should be screened by RT-PCR.
Mode and timing of delivery	Vaginal delivery does not increase the risk of neonatal infection. Cesarean section indicated if there is an imminent risk to the mother or baby [129,130]. No clinical trials have evaluated the effect of timing of delivery.	Mode of delivery should be guided as per obstetric indication. Delivery timing has to be individualized based on maternal health condition, co-morbidities and gestational age.
Protected rooming-in of newborn	Rooming-in with COVID-19-positive mother confers a higher risk of infection in the newborn [131]. Infection control measures can reduce the risk of postnatal transmission [132]. Benefits of rooming-in are based on the following (a) Rooming-in promotes exclusive breast feeding (b) Breastfeeding reduces neonatal mortality (c) Neonatal infection is mostly mild/asymptomatic.	Healthy newborns can be roomed in with COVID-19-positive mothers, with strict hygiene measures. Mother–baby dyad must be isolated from the uninfected cohort.
Breastfeeding	COVID registry data suggests no association between breastfeeding and infection transmission in newborns [133]. Antibodies are transferred to baby by breast milk and incidence of neonatal infection is lower in breastfed infants [131,134,135].	Exclusive breastfeeding (direct or expressed) is recommended for all newborn babies. Mothers must follow respiratory and hand hygiene measures while feeding and milk expression.
Pasteurization of milk	Holder’s method for pasteurization of donor human milk can inactivate the SARS-CoV-2 virus [136,137].	Milk Bank associations recommend that lactating women with COVID-19 disease should not donate breastmilk.
Face masks	High-quality evidence has shown that the use of N95 and surgical masks can reduce the incidence of infection (RR 0.43, 95% CI 0.29 to 0.64) [138,139]. Healthcare personnel, patients and attendants should follow CDC guidelines to maintain respiratory hygiene [120,140].	Healthcare workers (HCW) in non-COVID-19 areas should wear well-fitting face mask. HCWs in COVID areas should wear N95 masks, gowns, gloves, and eye protection. Face masks are not recommended for children < 5 years of age.
Hand hygiene	Hand hygiene recommendations are based on CDC guidelines [141].	HCW must perform hand hygiene using 60–95% alcohol-based hand rub or hand washing with soap and water for 20 s.
School closure/lockdown strategy	School closure prevented only 2–4% of deaths, which is less effective than other social distancing interventions [142,143]. School closure had a noticeable negative impact on education, psychosocial health, and economics [144,145,146].	School closure for reducing spread of COVID-19 disease should be among the last preventive measures and may be considered only in emergency situations.
Isolation of positive neonates	No randomized trials have compared the nursing of affected neonates in a shared NICU versus an isolated facility. Insufficient evidence of horizontal transmission from infected neonates [145,147].	Confirmed COVID-19 cases should be kept in separate areas designated in the hospital or intensive care unit.
Environmental disinfection	SARS-CoV-2 can be inactivated by surface disinfectants used in NICU, including chlorine-based disinfectants, detergents, alcohol, iodine-containing detergents, and hydrogen peroxide compounds [148,149,150].	Use 0.5% sodium hypochlorite to disinfect floors and walls. 70% ethyl alcohol for small surfaces between uses such as reusable dedicated equipment. Hydrogen peroxide can be used to clean incubators, infusion pumps, and ventilators when not in use.

## Data Availability

All data are available in this manuscript.
